# Laparoscopic sleeve gastrectomy in adults with Prader-Willi syndrome and super-morbid obesity: a case report and literature review

**DOI:** 10.3389/fendo.2026.1781163

**Published:** 2026-06-15

**Authors:** Ji Li, Linhua Hao, Yuan-Hui Jiang, Lei Chen

**Affiliations:** 1 Department of pharmaceutical preparation section, Qilu Hospital of Shandong University, Qingdao, China; 2 Research Center for Coastal Zone Science and Marine Development Strategy, First Institute of Oceanography, Ministry of Natural Resources, Qingdao, China; 3 Department of General Surgery, Qilu Hospital of Shandong University, Qingdao, China

**Keywords:** bariatric surgery, laparoscopic sleeve gastrectomy, multidisciplinary management, Prader-Willi syndrome, super-morbid obesity

## Abstract

**Objective:**

This study aimed to systematically assess perioperative clinical safety, medium-term weight loss outcomes, multidisciplinary management challenges, and the clinical applicability of laparoscopic sleeve gastrectomy (LSG) in adults with Prader-Willi syndrome (PWS) and super-morbid obesity (body mass index [BMI] ≥ 50 kg/m²), with the objective of identifying appropriate candidates for surgical intervention and establishing standardized perioperative management strategies.

**Case report:**

A 24-year-old male with PWS and super-morbid obesity (BMI: 64.44 kg/m²) underwent LSG following comprehensive multidisciplinary team (MDT) evaluation. Short-term postoperative recovery was uneventful; however, long-term weight reduction outcomes and the risk of postoperative complications remained uncertain. A review of the literature suggested that although LSG was associated with modest weight reduction in patients with PWS, significant postoperative management challenges remained, including behavioral issues, nutritional monitoring requirements, and the need for long-term multidisciplinary support.

**Conclusion:**

LSG should not be considered a first-line intervention for patients with PWS. Surgical intervention should be restricted to carefully selected patients following comprehensive MDT evaluation. Optimized perioperative behavioral management, together with emerging pharmacological therapies and non-invasive ventilatory support, may expand surgical eligibility within this patient population.

## 1 Introduction

Prader-Willi syndrome (PWS) is a rare genetic neurodevelopmental disorder caused by the loss of expression of paternally inherited imprinted genes within the chromosome 15q11–13 region. The disorder is characterized by hyperphagia, reduced metabolic rate, behavioral disturbances, and severe obesity (1). Conventional weight management approaches, including strict dietary regulation and structured physical activity programs, have demonstrated limited efficacy in individuals with PWS and are often difficult to sustain over the long term. In this context, bariatric surgical procedures such as laparoscopic sleeve gastrectomy (LSG) have been used for the management of morbid obesity in this population. The efficacy and safety of LSG in patients with PWS remain uncertain (2–4).

Alqahtani et al. reported that LSG was safe and effective in children and adolescents with morbid obesity associated with PWS, BBS, and other syndromic conditions, with substantial improvement in ghrelin levels observed among individuals with PWS (2). In contrast, a meta-analysis conducted by Goldstone et al. demonstrated no significant change in body mass index (BMI) from baseline among patients with PWS who underwent LSG (3). Chang et al. further reported postoperative complication rates ranging from 10% to 17%, with reoperation rates of approximately 7% following bariatric surgery in this population (4). Additional clinical evidence is required to clarify the role of bariatric surgery in this population and to adequately balance its potential benefits against associated risks. This report describes the clinical course of an adult patient with PWS and super-morbid obesity, defined as a BMI ≥ 50 kg/m², who underwent LSG. In addition, a review of the literature was conducted to assess the feasibility and challenges associated with this intervention and to support the development of individualized treatment strategies (5, 6).

## 2 Case report

### 2.1 Standardized baseline clinical data and unified diagnostic criteria

Super-morbid obesity in this case was diagnosed according to internationally accepted criteria, defined as a BMI ≥ 50 kg/m². The patient was a 24-year-old Han Chinese male who was admitted to the comprehensive weight management and bariatric surgery specialty outpatient department of the hospital with progressive and uncontrolled weight gain over a period of 6 years, accompanied by persistent hyperphagia, irresistible food-seeking behavior, intermittent daytime sleepiness, and fatigue for 5 years.

Standardized physical examination at admission demonstrated a height of 150 cm and a body weight of 145 kg, corresponding to a calculated BMI of 64.44 kg/m², meeting the diagnostic criteria for super-morbid obesity. Physical examination findings were notable for by severe central obesity, with extensive pale obesity-related abdominal striae. No abnormal dark-brown pigmentation was observed in skin folds, including the neck and axillary regions, thereby excluding the typical clinical manifestations of severe insulin resistance associated with acanthosis nigricans. A detailed medical history indicated that recombinant human growth hormone replacement therapy and adjunctive treatment had been administered during childhood and adolescence to improve body composition and physical development; however, treatment had been voluntarily discontinued at 18 years of age. Chromosomal genetic analysis performed in the hospital molecular genetics laboratory confirmed the classic deletion subtype of PWS involving the 15q11–q13 region, thereby establishing a definitive genetic diagnosis and excluding other syndromic forms of obesity disorders and simple severe obesity.

Comprehensive multidisciplinary team (MDT) assessment at admission identified multiple obesity-related comorbidities, including impaired glucose regulation with insulin resistance, mixed dyslipidemia characterized by elevated total cholesterol and triglyceride levels with reduced high-density lipoprotein levels, moderate non-alcoholic hepatic steatosis, and moderate obstructive sleep apnea-hypopnea syndrome (OSAHS). Complete documentation of baseline clinical characteristics and comorbidities was recorded in the electronic medical record system in accordance with standardized clinical case reporting requirements.

### 2.2 Standardized chronological clinical treatment course and supplementary perioperative parameters

First admission (June 3–7, 2025): Preliminary screening, suspected PWS, and preoperative weight reduction phase.

The patient was initially admitted because of severe obesity and associated metabolic dysfunction. Based on characteristic clinical manifestations, including pathological hyperphagia, a history of developmental delay, and behavioral abnormalities, PWS was strongly suspected. Given the potentially elevated perioperative risk associated with bariatric procedures in patients with undiagnosed syndromic obesity, the MDT elected to defer surgical planning and pending etiological confirmation through peripheral venous blood sampling and standardized chromosomal methylation analysis.

During the interval awaiting genetic testing results, standardized non-surgical weight reduction and risk optimization interventions were initiated. A personalized low-calorie, high-protein, and high-fiber structured dietary plan was developed by clinical nutrition specialists. Short-term administration of oral loop diuretics was implemented to improve fluid metabolism and reduce cardiopulmonary preload. Tirzepatide was administered subcutaneously for appetite suppression and metabolic optimization. Standardized nocturnal non-invasive positive-pressure ventilation was provided to improve nocturnal hypoxemia and reduce perioperative respiratory risk. Following 4 weeks of continuous standardized intervention, body weight decreased from 150 kg to 145 kg, resulting in improvement of the preoperative physical condition and reduction of the anticipated technical complexity associated with laparoscopic surgery.

Second admission (July 28–August 5, 2025): Genetic confirmation, MDT re-evaluation, and standardized LSG procedure.

Formal genetic testing confirmed a heterozygous deletion involving the chromosome 15q11–13 region with abnormal methylation findings, consistent with the deletion subtype of PWS. Subsequent MDT re-evaluation involving anesthesiology, respiratory medicine, endocrinology, clinical psychology, and nutrition was conducted. Comprehensive assessments of cardiopulmonary function, airway status for anesthesia, surgical tolerance, behavioral compliance, and nutritional status were completed. Absolute and relative contraindications to surgery, including severe cardiopulmonary insufficiency, uncontrolled behavioral or psychiatric crises, and severe malnutrition, were excluded. On August 2, 2025, three-dimensional high-definition LSG was performed under general anesthesia with endotracheal intubation by an experienced bariatric surgery team.

Key supplementary intraoperative parameters included maintenance of pneumoperitoneum pressure at 12–14 mmHg, estimated intraoperative blood loss of less than 20 mL, and a total operative duration of 58 minutes. No intraoperative adverse events, including visceral organ injury or major hemorrhage, were observed. Standardized postoperative adjunctive therapy included esomeprazole for prevention of stress-related ulceration and gastrointestinal mucosal injury, ursodeoxycholic acid for improvement of hepatic lipid metabolism and liver function protection, and routine supplementation with multivitamins, trace elements, and iron as part of postoperative nutritional management. Postoperative recovery was uneventful, and body weight at discharge on August 5, 2025, was 135 kg.

### 2.3 Standardized postoperative hierarchical follow-up, key efficacy outcome measures, and multidimensional metabolic outcomes

Short-term follow-up within 1 month after surgery: Primary wound healing of the abdominal surgical incision was achieved (grade A), with no postoperative complications observed, including incision infection, fat liquefaction, or delayed wound healing. At the 1-month outpatient follow-up assessment, body weight had decreased to 118 kg, corresponding to a cumulative absolute weight reduction of 17 kg. Quantitative efficacy assessment demonstrated a total weight loss percentage (TWL%) of 12.59% and an excess weight loss percentage (EWL%) of 28.36%, meeting the criteria for satisfactory short-term outcomes following bariatric surgery. Multidimensional metabolic assessment demonstrated significant improvement in overall nutritional status, with the lipid profile returning to the normal reference range. Follow-up abdominal color Doppler ultrasonography confirmed improvement of moderate hepatic steatosis to mild hepatic steatosis. Pre-existing mild iron-deficiency anemia did not worsen during the postoperative period. However, serum sex hormone levels and 25-hydroxyvitamin D levels remained below age-matched reference ranges, necessitating long-term targeted supplementation and dynamic monitoring.

**Figure 1:**
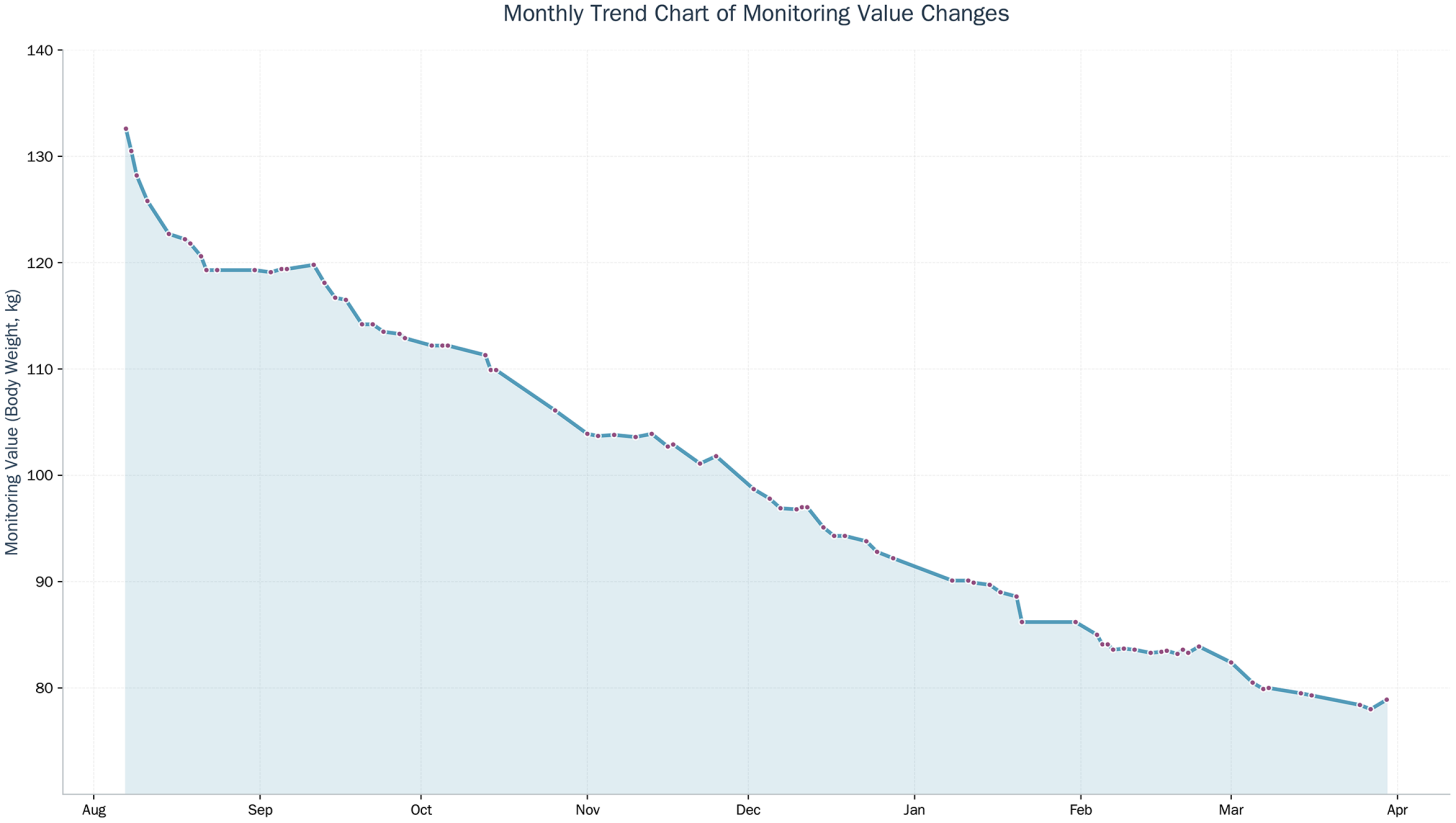
Postoperative weight-loss trajectory in a patient with PWS and super-morbid obesity following LSG. Monthly trends of key follow-up parameters are presented. The primary axis (blue line with circular markers) demonstrates progressive reduction in body weight (kg) throughout the 6-month follow-up period, indicating stable and sustained postoperative weight reduction without evidence of early weight regain.

Medium-term standardized management plan and 6-month follow-up outcomes: A dedicated MDT follow-up protocol was established, with scheduled evaluations at 3-month intervals to facilitate dynamic monitoring of body weight, nutritional and metabolic parameters, behavioral compliance, and psychological status. During the 6-month postoperative follow-up period after LSG, body weight decreased and stabilized at 86.2 kg, representing a cumulative absolute weight loss of 48.8 kg relative to discharge body weight. The calculated TWL% was 36.15%, and the EWL% was 65.72%. Detailed trends in body weight changes are presented in **Figure 1**. All medium-term metabolic outcomes were comprehensively documented, and no worsening or recurrence of obesity-related complications was observed during the follow-up period. At 6 months postoperatively, body weight had decreased by 48.8 kg, from 135 kg to 86.2 kg, as presented in **Figure 1**.

### 2.4 Complete genetic testing results and standardized imaging description

Molecular genetic analysis was performed using high-throughput chromosomal methylation-specific multiplex ligation-dependent probe amplification. A heterozygous deletion within the key pathogenic region of chromosome 15q11–13 was identified, accompanied by abnormal imprinting methylation resulting in loss of expression of paternally inherited genes within this region. These findings confirmed the diagnosis of the typical deletion subtype of PWS.

**Figure 2:**
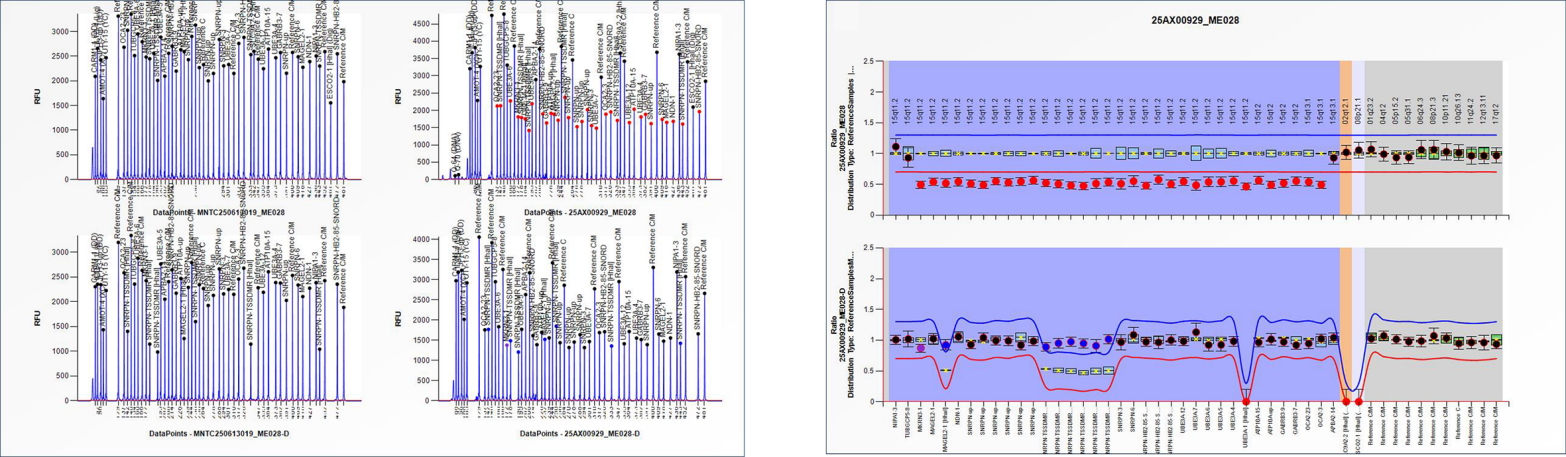
Comparison of fluorescence signal intensity profiles between the patient sample and the normal control sample (F202500421). Fluorescence signal intensity values ranging from 0.7 to 1.3 were defined as within the normal reference range.

Fluorescence signal intensity obtained from the patient sample was compared with that of a standard normal control sample from the institutional laboratory database (sample number: F202500421; **Figure 2**). The corresponding abnormal methylation peak patterns and comparative deletion analysis are presented in **Figure 2**. Standardized analytical criteria were applied uniformly, with fluorescence signal intensity values ranging from 0.7 to 1.3 defined as the normal reference range. Signal intensity values within the key genomic region were below the lower limit of the reference range, providing definitive genetic evidence supporting the diagnosis of PWS.

## 3 Literature review

### 3.1 Standardized literature retrieval methodology

A comprehensive literature search was conducted using the PubMed, Embase, Web of Science Core Collection, Cochrane Library, and China National Knowledge Infrastructure (CNKI) databases without restrictions on publication period to minimize temporal selection bias. The search strategy included the following English keywords: Prader-Willi syndrome, PWS, laparoscopic sleeve gastrectomy, LSG, bariatric metabolic surgery, super-morbid obesity, syndromic obesity, and multidisciplinary management, together with the corresponding professional medical terms in Chinese.

The inclusion criteria were as follows: (1) study population consisting of patients with genetically confirmed diagnosis of PWS and concomitant morbid or super-morbid obesity; (2) intervention involving LSG alone or in combination with adjunctive therapies; (3) study designs including clinical case reports, retrospective cohort studies, prospective observational studies, and authoritative meta-analyses; and (4) reported outcomes including perioperative safety, weight loss efficacy, incidence of complications, and postoperative management experience.

The exclusion criteria were as follows: (1) syndromic obesity disorders unrelated to PWS; (2) bariatric surgical procedures other than LSG used as the sole intervention; (3) *in vitro* studies, animal studies, review letters, and conference abstracts lacking complete data; and (4) duplicate publications with overlapping datasets.

Literature screening, quality assessment, and extraction of relevant data were independently performed by two clinicians. Discrepancies were resolved through consultation with a third senior specialist to ensure methodological rigor and objectivity throughout the review process.

### 3.2 Core pathophysiological characteristics and clinical management challenges of obesity in patients with PWS

Pathological hyperphagia as a core refractory mechanism: Hyperphagia represents the most prominent core clinical phenotype of PWS and is primarily attributed to congenital dysfunction of hypothalamic feeding centers and neural networks involved in appetite and energy regulation (7). Persistent excessive appetite, compulsive food-seeking behaviors, including food hoarding and food stealing, and other abnormal eating behaviors are commonly observed and contribute substantially to the progressive development of severe super-morbid obesity in this population. This central dysregulation of appetite differs fundamentally from increased appetite associated with environmental or behavioral factors in non-syndromic obesity, thereby limiting the effectiveness of conventional weight-loss approaches (7).

Congenital basal hypometabolism: Several clinical studies have demonstrated that basal metabolic rate in patients with PWS is approximately 15%–25% lower than that observed in healthy individuals and individuals with obesity of similar age, height, and weight (8). Even with long-term caloric restriction and structured exercise interventions, total energy expenditure remains relatively low, resulting in substantial difficulty in achieving sustained weight reduction and a high propensity for weight gain. Consequently, long-term management of obesity in this population remains challenging (8).

Complex behavioral and psychological cognitive comorbidities: Patients with PWS commonly exhibit mild-to-moderate intellectual developmental impairment, compulsive and stereotypic behaviors, emotional instability, anxiety, irritability, and cognitive rigidity (9, 10). These neurobehavioral characteristics may adversely affect adherence to long-term postoperative dietary recommendations, including low-sugar, low-fat, and high-protein dietary regimens, as well as lifelong vitamin and mineral supplementation. As a result, postoperative risks of nutritional deficiency, maladaptive eating behaviors, and weight regain may be increased in this patient population (9, 10).

High prevalence of multisystem obesity-related comorbidities: Adults with PWS and super-morbid obesity frequently present with multiple obesity-associated comorbidities, including moderate-to-severe obstructive sleep apnea, pulmonary hypertension, type 2 diabetes mellitus, atherosclerotic cardiovascular disease, and severe musculoskeletal degeneration (11). Endocrine-related complications such as congenital growth hormone deficiency, primary hypothyroidism, and progressive scoliosis are commonly observed, collectively contributing to increased perioperative anesthetic and surgical risks (11).

Reduced pain perception and delayed symptom recognition: Autonomic nervous system dysfunction has been reported in patients with PWS and may be associated with reduced pain sensitivity and delayed recognition of pain-related symptoms (12). This altered pain perception may obscure early clinical manifestations of postoperative complications, including gastric sleeve leakage, intra-abdominal hemorrhage, and intra-abdominal infection, potentially resulting in delays in diagnosis and treatment and contributing to an increased risk of postoperative mortality (12).

### 3.3 Theoretical clinical benefits of LSG in patients with PWS and super-morbid obesity

Restriction of functional gastric capacity: LSG is a standardized restrictive bariatric procedure, in which the majority of the gastric fundus and body along the greater curvature are resected, resulting in the formation of a narrow tubular gastric remnant. As a result, the effective physiological gastric capacity is substantially reduced, thereby physically limiting food intake and reducing excessive caloric consumption (13).

Reduction in peripheral ghrelin secretion: Ghrelin, a key appetite-stimulating hormone, is predominantly synthesized and secreted by the gastric fundus mucosa. Resection of the primary ghrelin-producing region during LSG may result in a substantial reduction in circulating ghrelin concentrations (14). Theoretically, this mechanism may partially attenuate abnormal hunger perception and has been considered one of the principal theoretical rationales supporting the use of LSG in patients with PWS and hyperphagia. Clinical cohort data reported by Fong et al. demonstrated a significant reduction in serum ghrelin levels within 3 months following surgery in patients with PWS (14).

Improvement of obesity-related metabolic comorbidities: Sustained weight reduction following successful LSG may contribute to improvement or partial resolution of multiple metabolic complications associated with long-term severe obesity, including insulin resistance, type 2 diabetes mellitus, mixed dyslipidemia, hypertension, and moderate obstructive sleep apnea (15). Findings reported by Chu et al. involving hepatic lipid metabolic pathways in severe obesity animal models proposed that effective weight reduction following LSG may reduce abnormal hepatic lipid deposition, thereby improving non-alcoholic fatty liver disease and potentially decreasing the long-term risk of metabolic liver disease (16).

Improvement in functional status and quality of life: Effective postoperative weight reduction may decrease mechanical stress on weight-bearing joints and improve cardiopulmonary function. Improvements in activities of daily living and reductions in dependence on caregivers may be observed. In addition, enhanced social functioning and psychological well-being may contribute to improved long-term quality of life outcomes (17).

### 3.4 Comprehensive evaluation of clinical efficacy and potential risks of LSG in patients with PWS

Core limitation: Incomplete control of central pathological hyperphagia: One of the principal limitations of LSG in patients with PWS is the inability of the procedure to fundamentally correct central pathological hyperphagia (18). Although peripheral ghrelin secretion may decrease following surgery, pathological hyperphagia in PWS is associated with complex dysfunction involving multiple hypothalamic neural regulatory pathways and therefore cannot be fully addressed through gastric volume reduction alone (18). Physical restrictions imposed by the procedure may be circumvented through the intake of high-calorie liquid or soft foods, while compulsive food-seeking behaviors, including food hoarding and food stealing, may persist. Consequently, risks of suboptimal weight reduction and long-term weight regain may remain elevated. Available clinical data have indicated that long-term weight reduction outcomes following LSG in patients with PWS may reach only 50%–70% of the efficacy observed in patients with non-syndromic obesity (14).

Increased perioperative risk associated with combined high-risk comorbidities: The coexistence of super-morbid obesity and OSAHS may substantially increase the complexity of anesthetic airway management and the risk of intraoperative cardiovascular and hemodynamic instability (7). Excessive abdominal adiposity and hepatic steatosis may increase technical complexity of laparoscopic procedures and elevate the risk of intraoperative visceral injury. Reduced pain perception may delay recognition of postoperative complications, while risks of deep vein thrombosis and pulmonary embolism may be increased (7). Aminian et al. reported elevated rates of postoperative respiratory and circulatory complications following bariatric surgery in patients with complex obesity-related conditions (19). Terekhin et al. further indicated that unique perioperative anatomical and physiological characteristics associated with rare genetic syndromes may contribute to increased perioperative risk in patients with PWS undergoing LSG (20).

Long-term compliance challenges and nutritional safety risks: Long-term postoperative adherence to high-protein, low-sugar, and low-fat dietary regimens may be challenging in patients with PWS. Persistent compulsive eating behaviors may increase the risk of postoperative gastric sleeve dilation, marginal leakage, and recurrent vomiting (18). Lifelong supplementation with vitamin B12, iron, calcium, and active vitamin D is required following bariatric surgery. However, limited long-term adherence may contribute to refractory nutritional anemia, osteoporosis, and multiple nutritional deficiencies (18). Continuous MDT follow-up is considered essential but may impose substantial long-term financial and caregiving burdens on patients and their families. In addition, dietary restriction may be associated with increased emotional distress, anxiety, and behavioral difficulties, thereby increasing the complexity of psychological management.

Risk of weight regain and ethical considerations: Long-term weight regain rates following LSG in patients with PWS have been reported to be higher than those observed in patients with non-syndromic obesity, with reduced durability of sustained weight reduction outcomes (21–23). Ethical considerations are important in this population as some patients with PWS may exhibit cognitive impairment that limits the ability to independently understand surgical risks, expected benefits, and lifelong postoperative management responsibilities. Accordingly, informed consent should therefore be obtained jointly with legal guardians when appropriate, and independent psychological and ethical assessments should be conducted to ensure appropriate ethical oversight and to minimize the risk of inappropriate surgical intervention.

### 3.5 Exploration of diversified alternative and complementary comprehensive therapeutic strategies

GLP-1 receptor agonist-based pharmacologic adjunctive therapy: Novel GLP-1 receptor agonists, including tirzepatide and semaglutide, delay gastric emptying and suppress central appetite signaling pathways, thereby demonstrating significant weight-reduction effects (13). Soomro et al. reported that combined treatment with GLP-1 receptor agonists and LSG in patients with PWS was associated with a 37.1% reduction in body weight within one year, accompanied by significant improvements in glucose metabolism and sleep apnea (13). Tong proposed that GLP-1 receptor agonists exert multiple regulatory effects on energy metabolism through GLP-1 signaling pathways and may contribute to optimization of postoperative metabolic outcomes (24). However, Li et al., Rejili et al., and Debnath et al. reported that GLP-1 receptor agonist therapy may be associated with adverse events including persistent nausea, vomiting, and in rare cases, gastric mucosal injury, indicating that individualized dose adjustment and safety monitoring are required during clinical use (25–27).

Alternative bariatric and metabolic surgical procedures: Alternative bariatric procedures, including Roux-en-Y gastric bypass (RYGB) and mini-gastric bypass may provide additional regulation of intestinal hormone secretion while restricting gastric capacity. Enhanced suppression of ghrelin secretion and stimulation of endogenous GLP-1 release may produce greater appetite-suppressive effects (28). Nevertheless, these procedures are associated with greater technical complexity, longer operative duration, greater perioperative risk, and a higher incidence rates of long-term nutritional complications and deficiencies. Consequently, their applicability in patients with PWS may be limited (28).

Foundational long-term multidisciplinary interventions: Long-term professional behavioral and psychological interventions, comprehensive family-based regulation of food access, structured supervision of daily routines, and regular supervised physical activity remain the cornerstone of obesity management in patients with PWS (29). Growth hormone therapy initiated during early childhood may improve body composition and physical development; however, its efficacy in controlling pathological hyperphagia remains limited and should not be considered a substitute for surgical or pharmacological interventions (29). Wang et al. proposed that multimodal adjunctive noninvasive interventions combined with family supervision may serve as a low-risk supplementary approach for the long-term management of metabolic disorders associated with PWS and may provide potential clinical benefit (30).

## 4 Discussion

This study presented a representative case of an adult patient with PWS and super-morbid obesity, combined with a standardized and rigorous literature review. Clear distinction was maintained between established medical evidence, findings derived from published literature, and individualized clinical observations from the present casein order to minimize conceptual ambiguity and avoid overgeneralization of conclusions derived from a single-case experience.

As a minimally invasive restrictive bariatric procedure, LSG should be regarded as a selective and individualized therapeutic option for adults with PWS and super-morbid obesity. Careful MDT-based decision-making throughout the preoperative period is essential. Gastric sleeve leakage has been reported as the most common serious complication associated with LSG (31). Due to persistent central pathological hyperphagia, excessive food intake may continue after surgery, thereby increasing the risk of acute gastric dilatation and potentially resulting in gastric wall necrosis or perforation with severe clinical consequences. Accordingly, LSG should not be considered a routine first-line intervention and should be restricted to carefully selected patients following comprehensive preoperative preparation and risk assessment. Miller et al. reported that although an initial short-term weight reduction effect may be achieved following LSG in patients with PWS, maintenance of long-term efficacy beyond 5 years may remain challenging (32).

Despite these limitations, the potential clinical benefits associated with selective surgical intervention should not be dismissed entirely. Progressive weight gain is a recognized component of the natural disease course of PWS (32). Obesity is a major contributor to morbidity and premature mortality in this population, and the average life expectancy of patients with PWS and uncontrolled severe obesity has been reported to be approximately 30 years shorter than that of individuals with normal body weight (33). Therefore, timely and appropriate early comprehensive intervention remains important for improving long-term clinical outcomes.

Current internationally accepted management of PWS involves stage-specific MDT-based comprehensive strategies, including long-term behavioral intervention, standardized hormone replacement therapy, and targeted management of obesity-related complications (34). Metabolic and bariatric surgery is recognized as one of the most effective clinical approaches for achieving sustained weight reduction in adults with severe obesity; however, its use in adolescents with PWS remains controversial (35, 36). For adults with PWS who demonstrate inadequate response to long-term conservative management, selective surgical intervention may provide substantial improvement in metabolic complications and quality of life, despite the potential risk of subsequent weight regain (37). Direct comparison of surgical outcomes between patients with PWS and patients with non-syndromic obesity may not be scientifically appropriate because the underlying pathophysiological mechanisms of obesity and patterns of postoperative weight regain differ substantially between these populations (38).

In the present case, the perioperative course was uneventful, and favorable short- and medium-term recovery outcomes were observed. However, long-term dynamic follow-up remains necessary. Although LSG involves resection of the principal site of ghrelin secretion, complete suppression of central pathological hyperphagia associated with PWS cannot be achieved through this intervention alone. In the present case, only mild residual hyperphagic behavior was observed, which may have been associated with comprehensive preoperative behavioral intervention and strict family supervision.

Perioperative combined risk management experience in the present case: Super-morbid obesity substantially increased the complexity of anesthetic and surgical management in this patient. Multidimensional preoperative interventions, including weight reduction, respiratory function optimization, and nutritional preparation, were implemented in combination with management by an experienced bariatric surgery team. These measures may have contributed to successful completion of the procedure without intraoperative adverse events.

Core challenges in long-term postoperative management: Behavioral non-adherence, binge-eating tendencies, and nutritional deficiencies represent common challenges in the long-term management of patients with PWS. In the present case, standardized MDT monitoring and active family supervision contributed to satisfactory postoperative dietary adherence during the medium-term follow-up, which may have contributed to favorable weight reduction outcomes.

Metabolic characteristics and implications for targeted intervention: Congenitally reduced basal metabolic rate represents an important factor contributing to difficulty in maintaining sustained weight reduction and to the increased risk of postoperative weight regain in patients with PWS. Therefore, continuous long-term follow-up is required to facilitate dynamic management of hyperphagic behaviors, nutritional status, and metabolic complications, thereby reducing the potential decline in long-term efficacy of restrictive bariatric interventions (39). Lifelong regular vitamin and mineral supplementation is required to reduce the risk of long-term complications, including nutritional anemia and osteoporosis.

Optimized future therapeutic strategies: Strengthened environmental regulation of food access and standardized behavioral interventions remain fundamental components of long-term management. GLP-1 receptor agonists represent potentially effective adjunctive therapies with relatively favorable safety profiles and may be used in combination with surgical intervention to enhance treatment outcomes and potentially reduce long-term surgical risks (40). Emerging melanocortin receptor-targeted therapies have also demonstrated potential utility in weight management among patients with PWS (41). Although RYGB may provide greater appetite regulation effects, concerns regarding the overall risk-benefit profile have limited its recommendation as a first-line therapeutic option (42). Growth hormone replacement therapy may improve body composition; however, severe hyperphagia and obesity are unlikely to be adequately controlled through this intervention alone (43–45).

Innovation and clinical implications of the present study: Previous studies have primarily focused on adolescents with PWS and obesity, whereas the present study specifically assessed an adult patient with super-morbid obesity, potentially increasing the relevance of the findings for this subgroup (21, 22). In the present case, preoperative optimization was achieved through combined administration of tirzepatide, noninvasive ventilatory support, and strict dietary intervention, resulting in a preoperative weight reduction of 12 kg and reduced surgical complexity. The case emphasized the importance of standardized perioperative MDT management and may provide useful clinical reference data for the management of similar future cases. Consistent with findings reported in the majority of published studies, the efficacy of LSG in patients with PWS appears to be limited and may be most appropriate for carefully selected patients with a BMI ≥50 kg/m², inadequate response to conservative management, and adequate family or caregiver support (32, 46). Although favorable outcomes were observed during the 6-month follow-up period in the present case, longer-term follow-up remains necessary to assess the durability of treatment efficacy.

## 5 Summary

1. No definitive curative treatment is currently available for PWS. Surgical interventions are primarily intended to manage refractory severe obesity and reduce obesity-related morbidity and mortality.
2. General anesthesia is associated with substantially increased perioperative risk in patients with PWS compared with the general population and therefore requires management by an experienced MDT.
3. Metabolic and bariatric surgery should not be considered a routine indication for patients with PWS and should be limited to carefully selected adult patients. LSG is associated with considerable perioperative risk and potentially limited long-term efficacy. Proposed selection criteria include a BMI ≥ 50 kg/m², inadequate response to standardized conservative treatment, and preservation of basic cognitive and behavioral compliance capacity.
4. Surgical decision-making should involve a comprehensive MDT, including specialists in endocrinology, bariatric surgery, psychology, nutrition, anesthesiology, and respiratory medicine.
5. Comprehensive perioperative management throughout the treatment course remains essential, with particular emphasis on continuous respiratory monitoring and individualized nutritional support, and long-term metabolic surveillance. Lifelong family supervision, regular behavioral intervention, and ongoing nutritional assessment remain important components of long-term postoperative management.
6. Future therapeutic strategies may include GLP-1 receptor agonists and emerging dual-target or multi-target anti-obesity agents, which may provide safer and more effective alternatives or adjunctive approaches to surgical intervention.

## 6 Conclusion

Selective metabolic and bariatric surgery may achieve rapid and effective medium-term weight reduction in carefully selected adults with PWS and super-morbid obesity and may reduce the short-term risk of severe obesity-related metabolic complications. However, the long-term efficacy and safety of this intervention require further evaluation through large-scale, multicenter prospective standardized clinical studies.

The development and implementation of a refined MDT-based perioperative management framework integrating minimally invasive bariatric surgery, behavioral and psychological interventions, individualized nutritional support, and targeted pharmacological therapy may contribute to improvement in the overall continuum of clinical care for patients with PWS and super-morbid obesity and may improve long-term clinical outcomes.

## Data Availability

The original contributions presented in the study are included in the article/supplementary material. Further inquiries can be directed to the corresponding authors.
